# Tamoxifen Promotes Axonal Preservation and Gait Locomotion Recovery after Spinal Cord Injury in Cats

**DOI:** 10.1155/2016/9561968

**Published:** 2016-02-23

**Authors:** Braniff de la Torre Valdovinos, Judith Marcela Duenas Jimenez, Ismael Jimenez Estrada, Jacinto Banuelos Pineda, Nancy Elizabeth Franco Rodriguez, Jose Roberto Lopez Ruiz, Laura Paulina Osuna Carrasco, Ahiezer Candanedo Arellano, Sergio Horacio Duenas Jimenez

**Affiliations:** ^1^Department of Computer Science, CUCEI, Universidad de Guadalajara, Avenida Revolucion No. 1500 Building M, Laboratory 212, 44430 Guadalajara, JAL, Mexico; ^2^Department of Physiology, CUCS Universidad de Guadalajara, Sierra Mojada 950, Building P Third Floor, 44290 Guadalajara, JAL, Mexico; ^3^Department of Physiology Biophysics and Neurosciences, Centro de Investigacion y Estudios Avanzados IPN, Avenida Instituto Politecnico Nacional 2508, 07360 Mexico City, DF, Mexico; ^4^Department of Veterinary and Medicine, CUCBA Universidad de Guadalajara, Camino Ing. Ramon Padilla Sanchez 2100, 45110 Zapopan, JAL, Mexico; ^5^Department of Neuroscience, CUCS Universidad de Guadalajara, Sierra Mojada 950, Building P Third Floor, 44290 Guadalajara, JAL, Mexico

## Abstract

We performed experiments in cats with a spinal cord penetrating hemisection at T13-L1 level, with and without tamoxifen treatment. The results showed that the numbers of the ipsilateral and contralateral ventral horn neurons were reduced to less than half in the nontreated animals compared with the treated ones. Also, axons myelin sheet was preserved to almost normal values in treated cats. On the contrary, in the untreated animals, their myelin sheet was reduced to 28% at 30 days after injury (DAI), in both the ipsilateral and contralateral regions of the spinal cord. Additionally, we made hindlimb kinematics experiments to study the effects of tamoxifen on cat locomotion after the injury: at 4, 16, and 30 DAI. We observed that the ipsilateral hindlimb angular displacement (AD) of the pendulum-like movements (PLM) during gait locomotion was recovered to almost normal values in treated cats. Contralateral PLM acquired similar values to those obtained in intact cats. At 4 DAI, untreated animals showed a compensatory increment of PLM occurring in the contralateral hindlimb, which was partially recovered at 30 DAI. Our findings indicate that tamoxifen exerts a neuroprotective effect and preserves or produces myelinated axons, which could benefit the locomotion recovery in injured cats.

## 1. Introduction

Thoracolumbar penetrating spinal cord injury (SCI) often produces motor and sensory alterations in hindlimbs [1]. Promising pharmacological treatments and treadmill locomotion training are used for inducing restoration of locomotion and spinal reflexes after contusive, compressive lesions or by a penetrating SCI [2–4]. Locomotion disturbances occur in concordance with the type of injury and the spinal cord area suffering the trauma [5, 6].

Axonal and neuronal death is an important secondary effect after a penetrating injury in brain and spinal cord lesions [7, 8]. Tamoxifen has been shown to be an effective treatment to brain and spinal cord injuries; it has been proposed as an inflammatory response modulator and participates in locomotion recovery after a SCI [2, 8, 9]. Tamoxifen is a selective estrogen receptor modulator (SERM) acting on *α*- and *β*-estrogen receptors; also, it is been shown to prevent demyelination and promote differentiation to oligodendrocytes from multipotential cells in the cerebral cortex [7]. It is unknown whether tamoxifen exerts neuroprotective effects in cats after a SCI.

In this study, experiments were made on cats with a T13-L1 level spinal cord hemisection and the effects of tamoxifen were assessed by evaluating the survival of neurons in the spinal cord, axonal myelin preservation, or remyelination and by analyzing the kinematic angular displacement of both hindlimbs during unrestricted gait.

## 2. Material and Methods

### 2.1. Subjects and Ethics Statement

Adult male cats were used in this study (Laboratory Animal Center of the Guadalajara University, 3.5–4 kg, *n* = 9). All the procedures described here were performed with the guidelines contained in the National Institutes of Health Guide for the Care and Use of Laboratory Animals (USA) and with the ethical considerations stipulated in the experimental animal treatment on the Official Mexican Norm (NOM-062-ZOO-1999). Experiments were approved by the ethics committee for research and biosafety (Universidad de Guadalajara).

### 2.2. General Procedures for Surgery and Spinal Cord Injury

Cats were divided in three groups: intact (INT, *n* = 3), injured and treated with tamoxifen (IWT, *n* = 3), and injured without tamoxifen (IWOT, *n* = 3). A prophylactic antibiotic treatment was given (Gentamicin 2 mg/kg i.m.). For preventing pain, Ketoprofen (2.5 mg/kg i.m.) was administered two days before the spinal cord hemisection. The cats were anesthetized with Ketamine (10 mg/kg i.p.) and Xylazine (1 mg/kg i.p.) for performing spinal cord injury. The dorsal surface of the T12 vertebra was exposed and the apophysis was removed with a surgical gouge. A microdrill was used for opening the right lamina, and a surgical blade (HERGOM® number 11) for hemisecting the spinal cord segment. After surgery, a drop of medical grade cyanoacrylate was applied on the dura mater and bone wax was used to cover the vertebra. Animals were treated for three days with postoperative antibiotics (Gentamicin 2 mg/kg i.m.). IWT cats received tamoxifen (1 mg/kg i.p.) at days 0, 1, and 2 DAI (days after injury). Animals had free access to water and food and were housed one per cage (1 m height × 1 m wide × 1 m tall) to allow them to move freely. Room temperature was maintained at 25-26°C.

### 2.3. Kinematic Analysis

**Figure 1:**
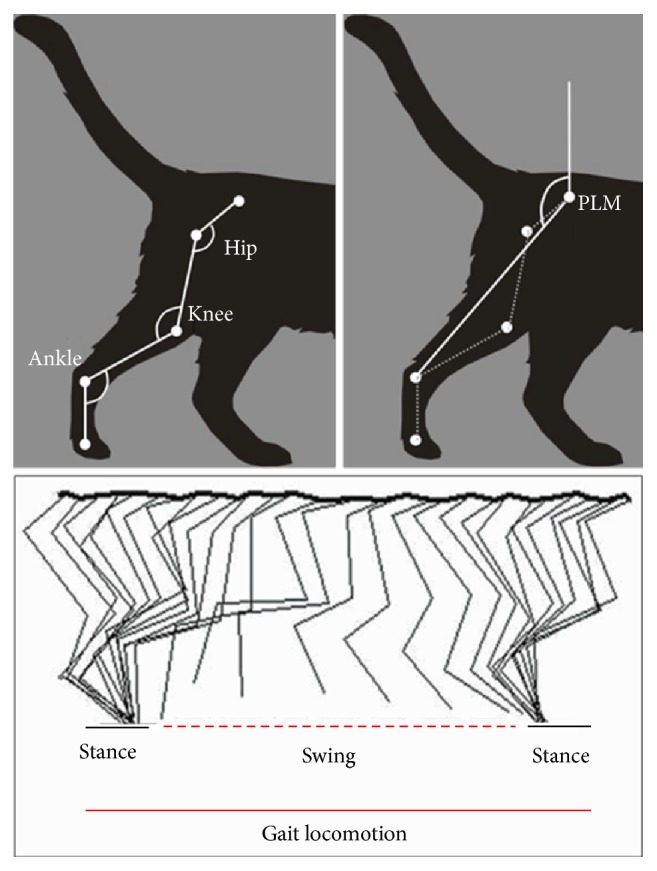
Diagram illustrating the experimental arrangement. Figure shows a schematic representation of the hip, knee, ankle, and pendulum like-movement (PLM) angles as well as the swing and stance step phases.

Prior to the surgery, animals were trained to walk through a transparent acrylic passway (200 cm long × 50 cm high × 20 cm width) daily during one week, in order to record a basal walk kinematic in cats. Video contrasting dots were placed in the iliac crest, hip (i.e., greater trochanter), knee, ankle (i.e., lateral malleolus), and fifth metatarsal phalangeal joints. The marks were placed in both hindlimbs and videotaped with a 30-frame-per-second video camera (SONY FDR-AX100). “Total video converter” (Shareware®) was used for decomposing the video into individual frames and the Cartesian coordinates of each joint mark were determined by the Image J software (Scion Corporation, NIH). Subsequently, the joint mark coordinates were introduced in a LabView® environment computer program (developed in CINVESTAV, IPN, México) [10]. Line graphs were constructed to illustrate the hindlimb movements from at least 3 consecutive strides. The computer program also calculates the hip and knee joint angles in a movement sequence executed by the ipsilateral and contralateral hindlimbs during strides. The joint angular displacement (JAD) was calculated from the difference between the maximum and minimal angular values of each stride. In addition, hindlimb PLM was analyzed by determining the angle between a line drawn from the iliac crest to the ankle and the *y*-axis (Figure 1). Control kinematics data was obtained prior to spinal cord injury in all subjects; subsequently, the experimental values were acquired at 4, 16, and 30 DAI in IWT and IWOT groups. All data were normalized and were graphically represented as a percent of the angular displacement.

### 2.4. Tissue Preparation

At 30 DAI cats were deeply anesthetized (pentobarbital euthanasia dose 50 mg/kg i.m.) and intracardially perfused with 500 mL of 0.1 M phosphate buffered saline (PBS) containing 0.5 mL of heparin (1000 U.I./mL), followed by 500 mL of a fixative solution (4% paraformaldehyde in 0.1 M PBS). The spinal cord segment T13-L1 was extracted and placed in the fixative solution during 24 hours and then washed in 0.1 M PBS for another 24 hours. The spinal cord segment was cryoprotected in 0.1 M PBS containing 30% of sucrose and 30% ethylene glycol for 72 hours. Subsequently, the spinal cord segment was divided in three regions: (1) injury site (IS), (2) cuts initiating 200 *μ*m rostral from the injury site (RFI), and (3) cuts initiating caudally from the injury site (CFI). The tissue was embedded in Leica® Tissue Freezing Medium and representative 15 *μ*m thick coronal cuts of each section were obtained from the T13-L1 segment using a Leica CM1850 cryostat.

### 2.5. Histology and Myelin Staining

Nine coronal cuts were placed per each slide; two slides per section were stained and analyzed. A Hematoxylin and Eosin (H&E) staining protocol was used for evaluating the histopathology status (observing whether Wallerian degeneration occurred; and the lesion similarity) of the spinal cord in INT, IWT, and IWOT cats. Hematoxylin solution was used for 5 minutes per slide and alcoholic Eosin Y solution (0.5% eosin in 90% ethanol) for 30 seconds; slides were washed with tap water for 5 minutes after staining. Toluidine blue staining protocol was used for assessing myelin thickness after SCI in all groups. O-Toluidine hydrochloride solution (3% o-toluidine in 0.1 M acetate-acetic buffer pH 5.0) was used for 5 minutes per slice; 2-minute washing was performed with tap water. Subsequently, an ethanol dehydration protocol was applied and the slices were mounted with Entellan®.

### 2.6. Axonal and Myelin Morphometric Analysis

The ventral white matter was visualized with light microscopy (Olympus, BX51W1) and photographed with a high resolution camera (Canon EOS Rebel T3). A 300 *μ*m^2^ square microscopic field was delimitated for establishing a qualitative white matter axon observation using the Portable Olympus® Image Pro plus software V 6.0. The myelin area was determined in axons from the ventral white matter (cat 1, *n* = 30; cat 2, *n* = 20; cat 3, *n* = 30, per analyzed section); the inclusion criterions were maximum diameter of 15 *μ*m and a round-like morphology. This study was performed using an image software analyzer (Motic Images Plus 2.0). The axonal myelin area was determined by establishing a perimeter trace around the outer myelin sheet and a second perimeter trace around the inside myelin sheet. The area between perimeters was calculated subtracting their respective area values.

### 2.7. Immunohistochemistry and Cell Count

Spinal cord tissue was pretreated with a PBS solution containing 0.3% X-100 Triton at 26°C for 30 minutes. The nonspecific antigen binding sites were blocked by a 10% normal goat serum for 1 hour at room temperature. The sections were incubated for 18 hours with the primary antibody at 4°C: anti-FOX3 for neurons, previously known as NeuN [11] (Abcam 104225, 1 : 1000). The slices were washed between incubations (three times) in PBS for 10 min. After primary antibody, the slices were incubated with the goat polyclonal secondary antibody: anti-IgG Alexa fluor 488 conjugated anti-rabbit (invitrogen A-11008, 1 : 1000) during 2 hours at room temperature. After the secondary antibody, tissues were incubated with 4,6-diamidino-2-phenylindole (DAPI) (Molecular Probes® D3571, 1 : 100) for 5 minutes. Slices were washed in PBS for 10 minutes. Nine coronal cuts per slide were placed manually, and three slides were evaluated in each level (cat 1: 18 cuts for RFI, 18 cuts for IS, and 18 cuts for CFI; cat 2: 18 cuts for RFI, 18 cuts for IS, and 18 cuts for CFI; cat 3: 18 cuts for RFI, 18 cuts for IS, and 18 cuts for CFI); the ethanol dehydration protocol was applied and sealed with Entellan. Neurons were counted using a digital image software (Portable Olympus Image Pro plus software V 6.0), adapted to a fluorescence microscope. Six microscopic fields (500 *μ*m^2^) were studied in the dorsal horn (DH; Rexed lamina I and II), ventral horn (VH; Rexed lamina VIII and IV), and periaqueductal zone (PAZ; Rexed lamina X), ipsilateral and contralateral to the injury. The total studied area was equivalent to 1.62 mm^2^.

### 2.8. Statistics

All data is expressed as mean ± SD. Neurons (*n* = 18 cuts per slide) and myelin morphometric (*n* = 30) data was analyzed using a nonparametric Kruskal-Wallis test and Mann-Whitney *U* test for multiple comparisons. Kinematic assessment was analyzed using a nonparametric Friedman test followed by Wilcoxon post hoc test for multiple specific comparisons. A *p* < 0.05 value was considered for establishing statistical significance. IBM SPSS (release 20.0.0) software was used for statistical tests and graphs were made using statistical software (GraphPad Prism 6.0).

## 3. Results

### 3.1. Tamoxifen Preserved the Contralateral Spinal Cord Tissue, in Similarly Injured Cats

**Figure 2:**
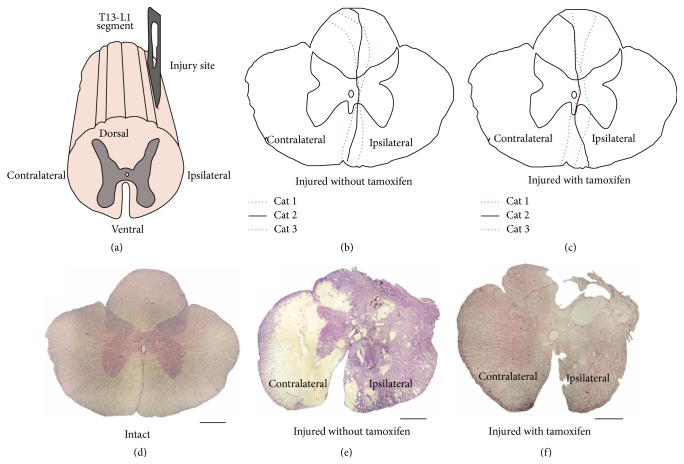
Representative T13-L1 microscopy images stained with H&E in coronal cuts (20 *μ*m thick) and schematic illustrations showing the lesion extension size. (a) Schematic diagram T13-L1 segment; (b) schematic diagram illustrating the lesion extension size for each one of the three cats injured without tamoxifen group; black continuous line represents cat 1, red circular dotted line cat 2, and blue oval dotted line cat 3; (c) schematic diagram showing lesions extensions in each one of the cats in the injured with tamoxifen group, lines as indicated. (d) Spinal cord coronal cut from an intact cat. (e) Spinal cord coronal cut from an injured cat without tamoxifen. (f) Spinal cord cut from an injured cat with tamoxifen; scale bar: 500 *μ*m.

A schematic drawing of the spinal cord damage for treated and untreated cats is illustrated in Figures 2(b) and 2(c). The spinal cord slices from intact cats show defined borders between gray and white matter. The gray matter showed a purple homogeneous color definition (basophilic staining, Figure 2(d)). Considerable damage, involving a large portion of the ipsilateral white and gray matter, is observed in the IWOT spinal cord tissue. The border between white and gray matter disappeared; the contralateral white matter showed hypochromic staining and the gray matter has poor basophilic and eosinophilic staining features showing abnormal characteristics (Figure 2(e)). In IWT cats, the ipsilateral side of the spinal cord has basophilic staining characteristics. Also tissue hallows as well as poor delimitation of white and gray matter were observed. In contrast, the contralateral side has preserved normal characteristics (Figure 2(f)).

### 3.2. Histological Analysis of White Matter Ventral Axons

**Figure 3:**
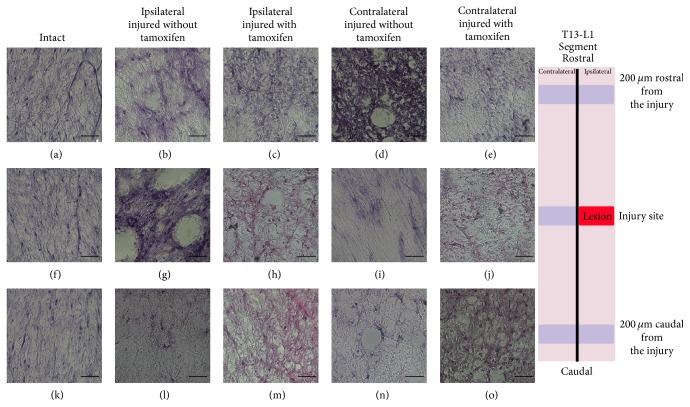
Illustrates ventral T13-L1 axons using H&E staining. ((a)–(o)) exhibits ventral axons of the white matter of the intact, injured treated with tamoxifen, and injured without tamoxifen cats in the ipsilateral and contralateral sides. ((a), (f), and (k)) Intact cat, ((b), (g), and (l)) ipsilateral side coronal cuts in an injured cat without tamoxifen, ((c), (h), and (m)) ipsilateral side coronal cuts from an injured cat treated with tamoxifen, ((d), (i), and (n)) contralateral coronal cuts obtained in an injured cat without tamoxifen, and ((e), (j), and (o)) contralateral coronal cut obtained from an injured with tamoxifen cat.

A qualitative assessment of the ventral white matter axons was made. Symmetrical axon morphology and a purple homogeneous color definition were observed in intact white matter, and there is no tissue inflammation or hollows (Figures 3(a), 3(f), and 3(k)). At the IS in the IWOT cats, axons in the ventral white matter of the ipsilateral side appeared with spheroid morphology (Figure 3(g)). In the contralateral side, a hypochromic staining with Wallerian degeneration characteristics was observed (Figure 3(i)). In IWT cats, axons appeared with a clearly defined morphology which was similar to INT cats axons at the ipsi- and contralateral side (Figures 3(h) and 3(j)). Similar normal stained characteristics were observed in CFI (Figures 3(l)–3(o)). At RFI, Wallerian degeneration was not observed in IWT or IWOT (Figures 3(b)–3(e)).

### 3.3. Tamoxifen Favored Ventral Axons Myelin Recovery of Injured Cats

**Figure 4:**
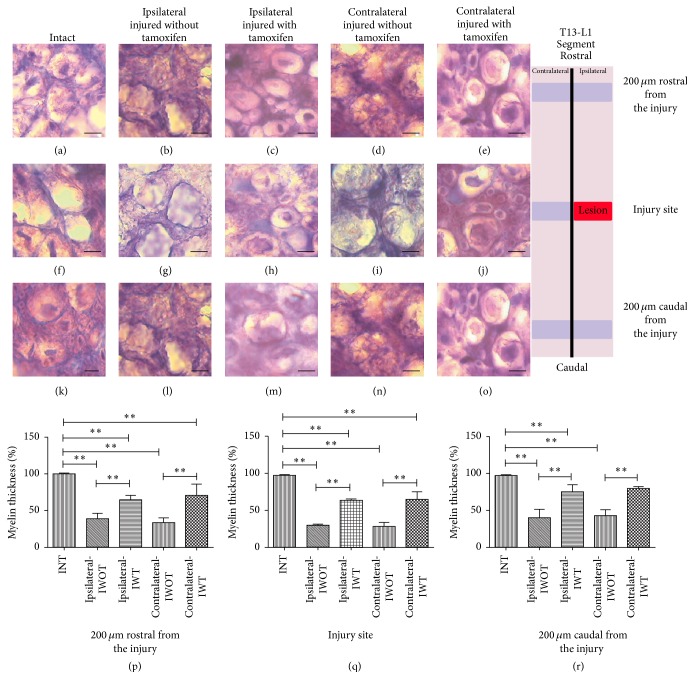
Myelin sheet of T13-L1 ventral axons is illustrated using Toluidine Blue staining. ((a), (f), and (k)) Axons in coronal cuts from an intact cat, ((b), (g), and (l)) ipsilateral axons from an injured cat without tamoxifen, ((c), (h), and (m)) ipsilateral axons from an injured cat with tamoxifen, ((d), (i), and (n)) contralateral axons in a cat without tamoxifen, and ((e), (j), and (o)) contralateral axons in a cat with tamoxifen. Graphs: ordinates exhibit the myelin thickness percentage from ventral zone axons; abscise different studied groups. (p) Myelin axon thickness valued at 200 *μ*m rostral from the injury, (q) axon myelin thickness valued in the injury site, (r) axon myelin thickness valued 200 *μ*m caudal from the injury situ, ^*∗∗*^ *p* < 0.001; scale bar: 5 *μ*m. Schematic diagram illustrates in site the rostral and caudal zones to value myelin thickness. The black horizontal line over the bars indicates a statistical significant difference between referred groups.

Myelin in INT, IWT, and IWOT ventral axons is illustrated in Figures 4(a)–4(o); intact axon myelin thickness in ventral axons was considered as 100%. Ipsilateral IS axonal myelin was reduced to 29.7 ± 2.4% in IWOT cats; in IWT cats, myelin was reduced to 64 ± 2.0% with a significant differences between groups (*p* < 0.001) (Figure 4(q)). Contralateral side myelin was reduced to 28.7 ± 6.7% in IWOT cats and to 65 ± 11.06% in IWT cats (*p* < 0.001). Similar results were obtained in the RFI and CFI regions (Figures 4(p) and 4(r), resp.). IWT and IWOT groups showed significant ipsilateral and contralateral statistical changes in the myelin percentages in comparison with INT cats. These changes occurred in IS, RFI, and CFI regions (Figures 4(p)–4(r)). Although the previously mentioned results were normalized to percentage values, real myelin thickness values are presented in the present work (Table 4).

### 3.4. Tamoxifen Effect on Neuronal Survival after Injury

**Figure 5:**
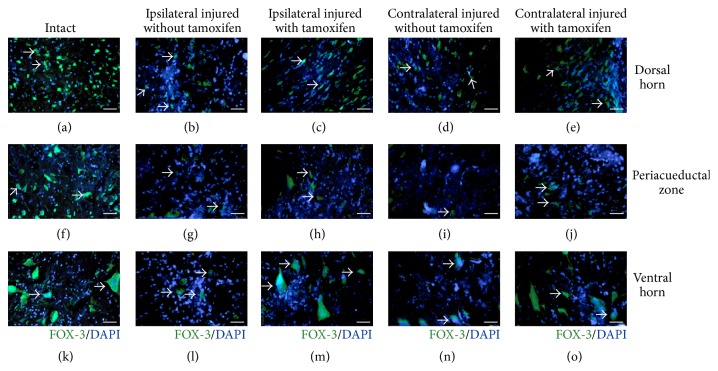
Microscopy images of FOX-3/DAPI positive cells in the dorsal horn, periaqueductal zone, and ventral horn, at T13-L1 spinal cord injury site. Dorsal horn neurons: ((b) and (c)) ipsilateral side, ((d) and (e)) contralateral side: (a) intact cat, (b) injured untreated cat, (c) injured treated cat, (d) injured untreated cat, and (e) injured treated cat. Periaqueductal Zone Neurons: ((g) and (h)) ipsilateral side and ((i) and (j)) contralateral side. (f) Intact cat, (g) injured cat without tamoxifen, (h) injured cat with tamoxifen, (i) injured cat without tamoxifen, and (j) injured cat with tamoxifen. Neurons in the ventral horn: ((l) and (m)) ipsilateral side and ((n) and (o)) contralateral side. (k) Intact cat, (l) injured cat without tamoxifen, (m) injured cat with tamoxifen, (n) injured cat without tamoxifen, and (o) injured cat with tamoxifen, scale bar 150 *μ*m; FOX-3 neurons in green, cell nuclei in blue.

**Figure 6:**
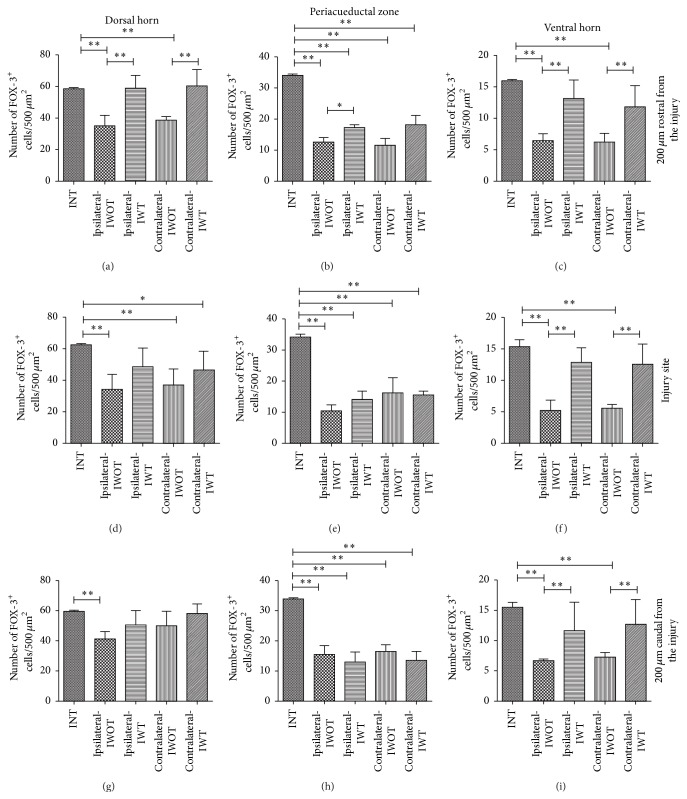
Number of counted neurons at the studied sites, graphs ordinates exhibited the number of FOX-3/DAPI positive cells and abscise different groups: intact, injured with tamoxifen, and injured without tamoxifen cats. Number of neurons 200 *μ*m rostral from the injury site: (a) dorsal horn, (b) periaqueductal zone, and (c) ventral horn. Number of neurons in the injury site: (d) dorsal horn, (e) periaqueductal zone, and (f) ventral horn. Number of neurons counted 200 *μ*m caudal from the injury: (g) dorsal horn, (h) periaqueductal zone, and (i) ventral horn; ^*∗∗*^ *p* < 0.001, ^*∗*^ *p* < 0.05. Schematic diagram illustrates rostral, injury site and caudal zones. The black horizontal line over bars indicates a statistically significant difference between referred groups.

FOX-3/DAPI positive cells were counted for evaluating spinal cord neuronal survival in INT, IWO, and IWT cats. At 30 DAI, an increase of the neuronal survival in tamoxifen treated cats was observed, particularly in the ventral zone (Figures 5(a)–5(r)). The number of neurons in INT, IWT, and IWOT groups in ipsilateral and contralateral side at the IS, RFI, and CFI regions is plotted in Figure 6. At 30 DAI, VH neurons decreased in IS, RFI, and CFI regions on both sides in IWT and IWOT cats. The number of ipsilateral and contralateral neurons was partially recovered by tamoxifen treatment (Table 1, Figures 6(c), 6(f), and 6(i)). Spinal cord damage reduced PAZ neurons in all studied regions, in treated and untreated cats (Table 1, Figures 6(b), 6(e), and 6(h)). DH neurons quantities did not change in the IS and the CFI site, in ipsilateral or contralateral spinal cord. This partial neuronal preservation occurred in treated and untreated cats (Figures 6(d) and 6(g)). It is important to mention that DH neurons were significantly reduced in the RFI region in untreated cats but recovered in treated cats in ipsilateral and contralateral spinal cord regions (Figure 6(a)).

### 3.5. Tamoxifen Treatment Induces a Recovery in the Hindlimb Gait Locomotion after Injury

**Figure 7:**
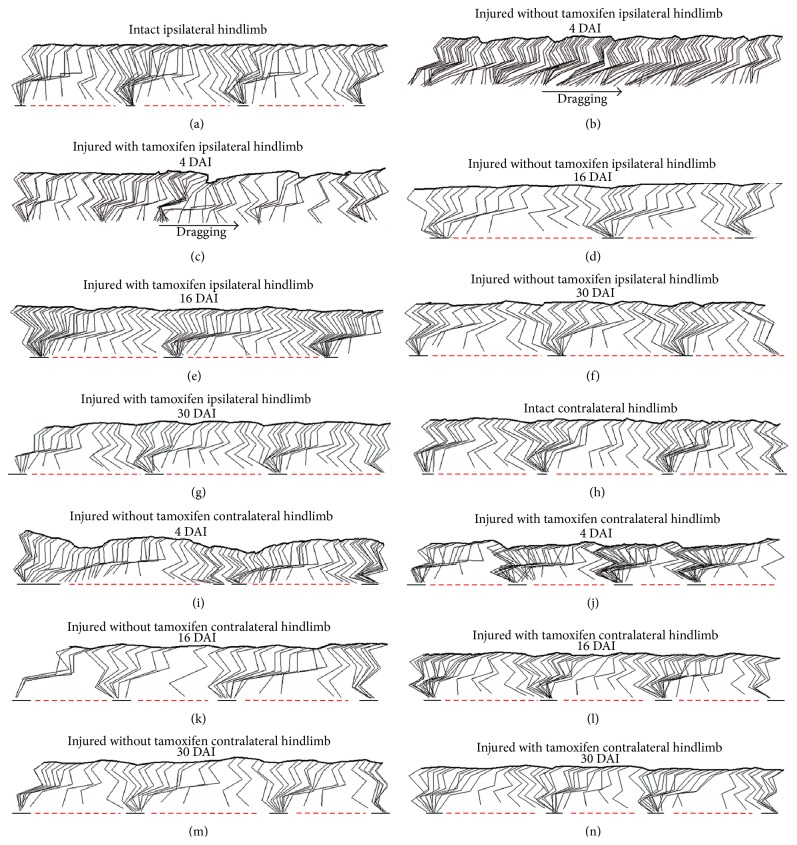
Stick figures illustrating both cat hindlimb during overground locomotion in intact and in spinal hemisected cats treated with tamoxifen. ((a)–(n)) Stick figures illustrating gait locomotion in intact cat and after 4, 16, and 30 DAI: in treated and untreated cats. (a) Ipsilateral hindlimb during locomotion in a intact cat. (b) Ipsilateral hindlimb during locomotion in an untreated cat at 4 DAI; arrow shows dragging during forward displacement. (c) Ipsilateral hindlimb locomotion in treated cat, at 4 DAI. (d) Ipsilateral hindlimb locomotion in an untreated cat, at 16 DP. (e) Ipsilateral hindlimb, in treated cat, at 16 DAI. (f) Ipsilateral hindlimb in an untreated cat at 30 DAI. (g) Ipsilateral hindlimb locomotion in treated cat, at 30 DAI. (h) Contralateral hindlimb locomotion in an intact cat. (i) Contralateral hindlimb locomotion in an untreated cat, at 4 DAI. (j) Contralateral hindlimb in treated cat, at 4 DAI. (k) Contralateral hindlimb locomotion in an untreated cat, at 16 DAI. (l) Contralateral hindlimb in treated cat, at 16 DAI. (m) Contralateral hindlimb locomotion in a untreated cat, at 30 DAI. (n) Contralateral hindlimb locomotion in treated cat, at 30 DAI.

**Figure 8:**
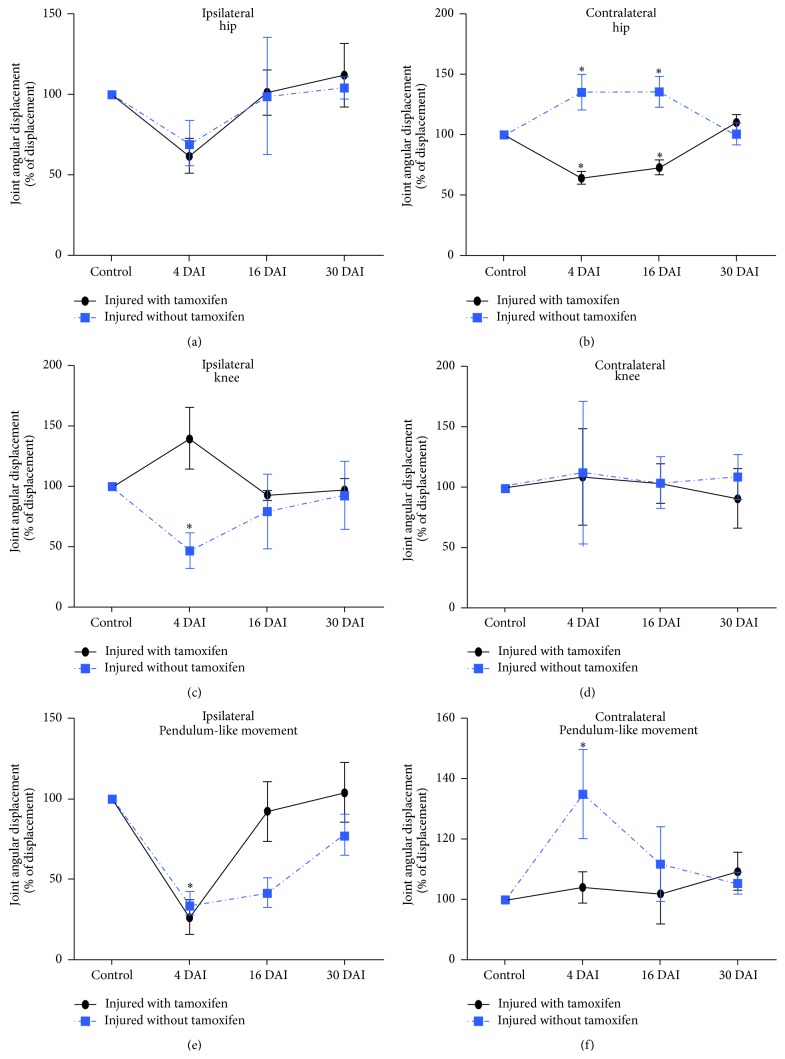
Graphs exhibit hip, knee joints, and the hindlimb pendulum-like movement angular displacement during stride. Movement changes in percent at different times (4, 16, and 30 DAI) comparing the respective angles and pendulum-like movement previous to the injury (control). ((a) and (b)) Hip angular displacement, ((c) and (d)) knee angular displacement, and ((e) and (f)) pendulum-like movement angular displacement; ^*∗*^ *p* < 0.05.

Gait locomotion was evaluated in IWT and IWOT cats before and after SCI. Three consecutive ipsilateral hindlimb (IHL) and contralateral hindlimb (CHL) strides were recorded (Figure 7). In normal stepping cats, the IHL and CHL executed symmetrical steps with well-defined stance and swing phases (Figures 7(a) and 7(h)). In contrast, at 4 DAI, IHL exhibited asymmetrical steps and limb dragging in both IWT and IWOT cats (Figures 7(b) and 7(c)). At 16 DAI, we observed that IWT and IWOT cats partially recovered their gait locomotor movements (Figures 7(d) and 7(e)). At 30 DAI, treated and untreated cats ILH showed a normal stepping sequence, indicating complete gait locomotion recovery (Figures 7(f) and 7(g)). At 4 DAI, IWOT cats exhibited an altered stride and oscillatory-like hip movements (as a compensatory process for maintaining gait locomotion and avoiding animal downfall) (Figure 7(i)). Stride alteration continued at 16 DAI (Figure 7(k)) and partial locomotion recovery is observed at 30 DAI (Figure 7(m)). In IWT cats, a locomotion recovery was observed at day 16 and it was clearly maintained until 30 DAI (Figures 7(l)–7(n)). The hip angular displacements in the IHL and CHL in IWT and IWOT cats are illustrated in Figure 8. Ipsilateral hip AD values in both groups of animals (IWT and IWOT cats) decreased approximately by 40% at 4 DAI but returned to their base values at 16 and 30 DAI (Figure 8(a) and Table 2). Whereas the IWOT cat contralateral hip exhibited a significant decrement in JAD values (nearly 50%) at 4 and 16 DAI, recovery was attained at 30 DAI. In contrast, the contralateral hip of IWT cats showed a considerable increment in JAD values (nearly to 150%; Table 2) at 4 and 16 DAI and recovered their base values at 30 DAI (Tables 2 and 3, Figure 8(b)). At 4 and 16 DAI, the ipsilateral knee of IWOT cats exhibited a statistically significant decrease in JAD values, approximately 50%, whereas the knee joint of IWT cats showed an increment in JAD values of approximately 50%. Both groups (IWT and IWOT cats) returned to their base values at 30 DAI (Figure 8(c)). No significant changes were observed in the contralateral knee (Tables 2 and 3, Figure 8(d)). In hindlimb PLM, both ipsi- and contralateral HAD values recovered faster in cats treated with tamoxifen, as compared to nontreated animals. At 4 DAI, IHL angular displacement values showed a statistically significant decrease in both, IWT and IWOT groups. IWT cat's pendulum-like movement values showed partial recovery at 16 DAI and nearly a 100% recovery at 30 DAI. At 30 DAI, pendulum-like movement of IWOT cats recovered by nearly 90% (Tables 2 and 3, Figure 8(e)). At 4 and 16 DAI, IHL angular displacement values in IWOT cats increased by 50% and 20%, respectively. At 30 DAI, angular displacement values were nearly recovered to 100%. In IWT cats, no differences occurred in the contralateral PLM (Tables 2 and 3, Figure 8(f)).

## 4. Discussion

The penetrating injury applied in our model produced a degeneration process characterized by spheroid axons and tissue hollows occurring in ipsilateral as well as in the contralateral side of the injury. A Wallerian degenerative process seems to be occurring on both sides in accordance with previous results, reporting an axonal Wallerian degeneration after a spinal cord injury [12, 13].

Tamoxifen is SERM acting on the *β*-estradiol estrogen receptors (ER) [14, 15]. It has clinical therapeutic uses in human breast cancer treatment. In addition, it produces neuronal protection after brain penetrating injury [16] and reduces microglia reactivity [17]. In rats, tamoxifen reduces the inflammatory process after a spinal cord injury [8] and the neuronal death [9]. These effects could be attributed to its actions on glial and neuronal ER. Further experiments studying tamoxifen's effects on neural inflammation process, neurotrophic factors, and inflammatory interleukins would be required for comparing tamoxifen's effects in cats to those observed in rats.

Contusion impact in a cat's spinal cord produces an altered axonal morphology [18]. In our study, tamoxifen reduced spheroid axons in the ipsilateral spinal cord in IWT cats, recovering a quasi-normal morphology. The contralateral axons in the IWT group were partially preserved. This preservation could be favored by an inhibition of the glutamate excitotoxicity after the injury and also by an attenuation of inflammatory mediated damage [8, 19].

Axonal myelin and cytoskeletal proteins degenerate after a SCI [20]. Our results reveal a myelin loss in IWOT cats. In IWT animals, partial myelin preservation occurred. However, it is unknown whether myelin preservation was due to remyelination. Previous works indicate that tamoxifen promotes NG2 multipotent progenitor cell differentiation changing into oligodendrocytes and could favor remyelination after rat brain penetrating injury [7, 21]. Tamoxifen also spared mature oligodendrocytes after rat SCI favoring remyelination [2].

Rat spinal cord contusion produces deleterious effects in neurons at 24 hours [22, 23]. After one month, they were attributed to an inflammatory process. In IWOT cats, at 30 DAI, after the spinal cord injury, the number of positive FOX-3 cells was reduced on both ipsilateral and contralateral sides. In IWT cats, the number of neurons in VH and DH is similar to the number in INT cats. This result could be related to the morphological ER distribution in the thoracolumbar spinal cord, as reported in other animal species [24–26]. The number of neurons in the PAZ did not recover after treatment. This effect could be attributed to a minor amount of ER of the PAZ neurons. It has been demonstrated that estrogen receptors are poorly expressed in periaqueductal neurons and are present mostly in the ventral horn in the female cat's lumbosacral spinal cord [27]. Further experiments must be performed for evaluating the spinal cord ER distribution in male cats.

PAZ neurons are involved in locomotion alternation [28]. Therefore, important contralateral plastic changes could be contributing to a rapid locomotion recovery but not through the PAZ neurons pathway.

In this study we analyzed overground locomotion. At 16 DAI, tamoxifen recovered the hindlimbs AD in walking cats. In adult cats, spinal cord injury models had been previously described [29–31]. In these models, the locomotion recovery was related to the severity of the SCI. In the present experiments, all cats suffered a similar damage at the T13-L1 level (Figure 2). Therefore, the locomotion recovery by tamoxifen treatment was consistent because the damage of the spinal cord extension was similar in all cats. In the present experiments, the locomotion onset might depend more on the undamaged contralateral spinal cord than a step training plasticity process, given that the cats were not trained for walking.

The kinematic parameters were altered during the first 16 DAI in both hindlimbs but were partially or fully restored after 30 DAI. It is of interest that, in IWT cats, recovery to nearly normal values already occurred at 16 days; therefore tamoxifen shortened the recovery time. To the best of our knowledge, hemisected cats treated with tamoxifen and walking overground were studied for the first time. Locomotion recovery results observed in this study highlight the importance of preserving contralateral descending pathways for locomotion initiation and kinematic recuperation [32]. It would be of interest to study the effects of tamoxifen in relation to avoiding obstacles during a cat's normal walking task. A previous study sheds light on this question, in which they assessed hemisected spinal cord cats during overground walking while avoiding obstacles in their way [33].

In addition to what was previously stated, a contusion model in rats has been used for studying tamoxifen anti-inflammatory effects, but the kinematics in injured rats under tamoxifen treatment remain to be established [5, 34].

At present time, we consider that a study must be made in which the combined treatment with tamoxifen and treadmill training to improve the outcome of hemisected animals should be carried out.

## 5. Conclusions

The current study demonstrates that hemisected spinal cord produces important locomotion alterations in both hindlimbs. Tamoxifen has important effects on axonal and myelin preservation, favoring cat locomotion recovery. The drug has been previously tested in the murine species with positive neuroprotective effects. This study shows beneficial outcomes in a repertory of motor patterns. Tamoxifen may be useful in several animal species as therapeutic treatment in spinal cord injury.

## Figures and Tables

**Table 1:** Number of FOX-3/DAPI positive cells.

Site	Ipsilateral									Contralateral								
	INT			IWOT			IWT			INT			IWOT			IWT		
	DH *n* = 18	PAZ *n* = 18	VH *n* = 18	DH *n* = 18	PAZ *n* = 18	VH *n* = 18	DH *n* = 18	PAZ *n* = 18	VH *n* = 18	DH *n* = 18	PAZ *n* = 18	VH *n* = 18	DH *n* = 18	PAZ *n* = 18	VH *n* = 18	DH *n* = 18	PAZ *n* = 18	VH *n* = 18
200 *µ*m RFI	59 ± 1	33.8 ± 1.4	15.9 ± 0.1	34.7 ± 6.3	12.5 ± 2.1	6.3 ± 1.2	58.7 ± 8	17 ± 1.6	13.1 ± 3	58 ± 0.5	32.1 ± 1.3	15 ± 1	38.5 ± 2.3	4 ± 2.3	6.2 ± 1.5	60.4 ± 10.2	4.9 ± 2.8	11.8 ± 3.4

IS	62.3 ± 0.8	32.4 ± 1.2	15.4 ± 1.1	33.6 ± 10.5	10.3 ± 2.4	5.3 ± 1.7	48.5 ± 11.9	13.72 ± 4.4	12.8 ± 2.5	63.1 ± 1	31.4 ± 1.6	16 ± 0.8	39.9 ± 10.4	16 ± 8.4	5.5 ± 0.6	46.2 ± 12	14.9 ± 2.5	12.6 ± 3.1

200 *µ*m CFI	58.6 ± 1.2	33.5 ± 1.1	16 ± 0.4	41.1 ± 4.4	15.5 ± 5.3	6.7 ± 0.7	50.3 ± 9.5	13.1 ± 6.1	11.7 ± 4.7	61 ± 0.6	33.1 ± 2	17 ± 1	50.1 ± 8.8	16.4 ± 4	7.2 ± 0.8	57.8 ± 5.5	13.4 ± 4.7	12.7 ± 4

INT, intact; IWOT, injured without tamoxifen; IWT, injured with tamoxifen; DH, dorsal horn; PAZ, periaqueductal zone; VH, ventral horn; RFI, rostral from injury; IS, injury site; CFI, caudal from injury.

**Table 2:** Comparison of the ipsilateral hindlimb angular displacement to control during locomotion at 4, 16, and 30 DAI.

	IHL												
Joint	Control	4 DAI				16 DAI				30 DAI			
	INT	IWOT	*p* value	IWT	*p* value	IWOT	*p* value	IWT	*p* value	IWOT	*p* value	IWT	*p* value
Hip	100	69.8 ± 14.5	NS	62.2 ± 10.8	NS	99.0 ± 36.2	NS	101.3 ± 14	NS	104.1 ± 7.1	NS	112.1 ± 20	NS
Knee	100	46.6 ± 14.7	^*∗*^ *p* < 0.05	140.3 ± 25.8	NS	80.2 ± 3.8	NS	92.6 ± 3.3	NS	92.7 ± 28	NS	98 ± 8.9	NS
Ankle	100	106.8 ± 35.8	NS	155.5 ± 47.8	^*∗*^ *p* < 0.05	131.3 ± 19	NS	92.23 ± 31	NS	138.3 ± 16.1	NS	94.7 ± 22.3	NS
PLM	100	35.2 ± 7	^*∗*^ *p* < 0.05	26.7 ± 10.1	^*∗*^ *p* < 0.05	41.4 ± 9.6	^*∗*^ *p* < 0.05	92.8 ± 18.3	NS	77.6 ± 12.8	NS	104 ± 18.9	NS

LH, ipsilateral hindlimb; NS, not significant; INT, intact; IWT, injured with tamoxifen; IWOT, injured without tamoxifen; DAI, days after injury; *∗* denotes *p* < 0.05.

**Table 3:** Comparison of the contralateral hindlimb angular displacement to control during locomotion at 4, 16, and 30 DAI.

	CHL												
Joint	Control	4 DAI				16 DAI				30 DAI			
	INT	IWOT	*p* value	IWT	*p* value	IWOT	*p* value	IWT	*p* value	IWOT	*p* value	IWT	*p* value
Hip	100	135.5 ± 14.2	^*∗*^ *p* < 0.5	64.09 ± 5.07	^*∗*^ *p* < 0.05	135.8 ± 12.8	^*∗*^ *p* < 0.05	73.2 ± 6.1	^*∗*^ *p* < 0.05	100.7 ± 8.6	NS	110.5 ± 6.5	NS
Knee	100	112.5 ± 59.2	NS	109.1 ± 39.9	NS	103.8 ± 21	NS	103.6 ± 16.5	NS	108.9 ± 18.8	NS	90.96 ± 24.8	NS
Ankle	100	140.7 ± 18	NS	127.3 ± 41.3	NS	149.7 ± 53.2	NS	105.6 ± 36.5	NS	180.7 ± 80.6	NS	99.3 ± 18.5	^*∗*^ *p* < 0.05
PLM	100	135.25 ± 14.8	^*∗*^ *p* < 0.05	104.23 ± 5.1	NS	112 ± 12.5	NS	102.1 ± 10.1	NS	105.7 ± 3.6	NS	109.6 ± 6.3	NS

CHL, contralateral hindlimb; NS, not significant; INT, intact; IWT, injured with tamoxifen; IWOT, injured without tamoxifen; DAI, days after injury; *∗* denotes *p* < 0.05.

**Table 4:** Myelin thickness in micrometers.

	Ipsilateral						Contralateral					
	INT *n* = 30	IWT *n* = 30	IWOT *n* = 30	INT vs IWT	INT vs IWOT	IWOT vs IWT	INT *n* = 30	IWT *n* = 30	IWOT *n* = 30	INT vs IWT	INT vs IWOT	IWOT vs IWT
200 *µ*m RFI	39 ± 11.3 *µ*m	22 ± 5.7 *µ*m	13.7 ± 5.8 *µ*m	*p* < 0.001	*p* < 0.001	*p* < 0.001	38 ± 5.6 *µ*m	23.5 ± 8 *µ*m	10.8 ± 4 *µ*m	*p* < 0.001	*p* < 0.001	*p* < 0.001
IS	40 ± 10.9 *µ*m	21.8 ± 6 *µ*m	10.5 ± 3.6 *µ*m	*p* < 0.001	*p* < 0.001	*p* < 0.001	41 ± 6.4 *µ*m	22.5 ± 9 *µ*m	9 ± 4.6 *µ*m	*p* < 0.001	*p* < 0.001	*p* < 0.001
200 *µ*m CFI	39 ± 9.8 *µ*m	25.8 ± 9 *µ*m	13.7 ± 6.5 *µ*m	*p* < 0.001	*p* < 0.001	*p* < 0.001	43 ± 2.6 *µ*m	29 ± 8 *µ*m	14 ± 4.2 *µ*m	*p* < 0.001	*p* < 0.001	*p* < 0.001

INT, intact; IWOT, injured without tamoxifen; IWT, injured with tamoxifen; RFI, rostral from injury; IS, injury site; CFI, caudal from injury.
